# Quality improvement of community pharmacy services: a prioritisation exercise

**DOI:** 10.1111/ijpp.12354

**Published:** 2017-03-27

**Authors:** Rumana S. Newlands, Ailsa Power, Linda Young, Margaret Watson

**Affiliations:** ^1^ Health Services Research Unit University of Aberdeen Foresterhill Aberdeen UK; ^2^ NHS Education for Scotland Glasgow UK; ^3^ NHS Education for Scotland Dundee Dental Education Centre Dundee UK; ^4^ Department of Pharmacy and Pharmacology University of Bath Bath, UK

**Keywords:** community pharmacy services, Delphi technique, health care research, knowledge translation, quality improvement

## Abstract

**Objectives:**

Effective strategies are needed to translate knowledge (evidence) into practice to improve the quality of community pharmacy services. We report the first step of a novel knowledge translation process which involved the systematic identification and prioritisation of community pharmacy services in Scotland which were perceived to require improvement and/or guideline development.

**Methods:**

This process involved three stages and a stakeholder group comprising community pharmacists, policy makers, lay and pharmacy organisation representatives. A modified nominal group technique (NGT) was used for topic generation (August 2013) followed by an electronic Delphi survey (eDelphi), October–December 2013) and topic rationalisation (December 2013) based on feasibility, acceptability, and potential impact for practice improvement.

**Key findings:**

In total, 63 items were identified during the modified NGT which were categorised into 20 topics to form the starting point of the eDelphi. In total, 74 individuals (mostly community pharmacists) indicated an interest in the eDelphi, which achieved response rates of 63.5%, 67.6%, and 70.3%, respectively in Rounds 1, 2, and 3. Consensus was achieved with six topics: promoting the appropriate sale and supply of over‐the‐counter medicines; patient counselling for prescribed medication; pharmaceutical care to promote medication adherence; promotion and delivery of a Minor Ailment Scheme; pharmaceutical care of vulnerable patients; and effective use of community pharmacy workforce. Of these, the priority topic selected for the next stage of the programme was *promoting the appropriate sale and supply of over‐the‐counter medicines*.

**Conclusions:**

This study adopted a systematic, inclusive, and rapid approach to identify priorities for community pharmacy practice improvement in Scotland.

## Introduction

The delivery of safe and high‐quality health care services is challenging,^1^, ^2^ and the translation of research findings into practice is inconsistent.^2^, ^3^ Obstacles to implementation can arise at multiple levels of health care delivery: patient, provider, policy, and/or the larger system or environment in which the organisations are embedded.^4^, ^5^, ^6^ The critical role of implementation research (also referred to as knowledge translation and/or improvement science) is gaining increasing recognition with health service researchers wishing to translate research findings into meaningful patient care outcomes.^7^, ^8^, ^9^

National Health Service (NHS) Education for Scotland (NES) offers a wide range of education and training support for clinical and non‐clinical staff who work in National Health Service (NHS), Scotland.^10^ Since 2008, NES has funded a major, national programme called ‘Translation Research in a Dental Setting (TRiaDS)’.^11^ The TRiaDS programme has established a practical evaluative framework for the translation of guidance through the conduct of a multidisciplinary programme of translation research.^11^ This programme supports the three quality ambitions: safe, effective, and person‐centred care.^12^ It involves a multidisciplinary team comprising health care professionals, policy makers, guidance developers, and international experts from a range of research disciplines such as health economics, health psychology, and health services research. Due to the success of TRiaDS, NES expanded the research programme to include community pharmacy and community optometry in 2013. The TRiaDS approach was used to meet the overall aim of the TRiaDS in Pharmacy (TRiaDS‐P) programme, i.e. to translate research findings into meaningful patient care outcomes. Unlike community dentistry in Scotland, there are no specific clinical guidelines for community pharmacy practice. Therefore, the aim of this study was to systematically identify and prioritise community pharmacy services in Scotland which required improvement and/or guideline development.

## Method

### Design of the study

The prioritisation study was undertaken as a three‐stage process involving a modified nominal group technique (NGT)^13^, ^14^ with key stakeholders; an electronic Delphi (eDelphi) survey; and rationalisation of priority topics. The NGT and Delphi are commonly used in pharmacy practice research to achieve consensus.^15^ A modified NGT was used in this current study to generate topics in a fast and efficient manner during a face‐to‐face meeting.

### Stage 1: key stakeholders: topic generation using the nominal group technique

Key stakeholders, representing the Royal Pharmaceutical Society in Scotland and the Scottish Government's Pharmacy & Prescribing Support Unit, were identified through professional and personal networks. A face‐to‐face stakeholder meeting was held in August 2013. The meeting comprised a brief introduction to the purpose and goals of the event followed by a modified NGT to identify priority topics.

The modified NGT involved each participant independently generating topics in response to the question ‘What do you think are the priority topics/areas for community pharmacy practice improvement’. Each participant contributed one topic to each round of the process until topic generation was exhausted. No discussion was permitted during this stage; only clarification of the meaning of the topic was sought if necessary. This process maximised the number of topics generated and increased the richness of the data collected.^16^, ^17^ The key stakeholders then discussed the topics and formulated them into common themes.

### Stage 2: the Delphi survey

The purpose of the Delphi process^18^ was to develop consensus amongst a wider group of stakeholders regarding the topics generated during Stage 1. An eDelphi was conducted using the NES portal and Questback (https://www.questback.com/uk/) to facilitate timely response. Three rounds were undertaken.

#### Participants and recruitment

Delphi panel members were identified using two methods. Firstly, research team and Professional Advisory Group members generated a list of senior community pharmacists, representatives from pharmacy organisations, policy makers, academics, and researchers (*n* = 31). The members of the key stakeholder group were also eligible to participate. The second method involved an email invitation being sent via the NES portal to all community pharmacists who had previously attended an NES training event (2342 individuals of whom 1450 were ‘community pharmacy employees’, 487 were community pharmacy locums, and 203 were community pharmacy owners). Recipients were requested to respond to the invitation if they were willing to participate in the eDelphi.

#### Process of determining priorities

The topics generated in Stage 1 formed the basis of the first eDelphi round and were presented in random order. Participants were asked to rate the importance of each topic as a priority for guideline development and/or practice improvement using a 7‐point Likert scale (1 = not important at all to 7 = extremely important). Likert points 2–6 were unlabelled, i.e. they were presented in a numerical format only. Demographic data were collected including sex, age, NHS board, employment status, type of pharmacy sector, type, and size of pharmacy. During this first round, respondents could add new topics to the existing list if they considered them to be priorities. Anonymised results from each round were fed back to the entire cohort for the subsequent rounds with a 2‐week deadline for responses. The median score from each round was presented alongside each topic to provide participants in Rounds 2 and 3 with information about the collective opinion. An email reminder was sent for Rounds 2 and 3 one week prior to the deadline along with a link to the survey to maximise response rates. In Round 1, consensus was defined using the median score with topics that achieved a median score >5 for inclusion in Round 2. For Rounds 2 and 3, an a priori decision was made to invoke a cut‐off of the 25th percentile, i.e. ≥6, for eligibility for inclusion in the subsequent round/final priority list. In Round 3, participants were asked to indicate whether national guidance was available on the topic at the time of the survey and provide details.

#### Analysis

Summary statistics (mean, standard deviation, median, and interquartile range) were calculated for topics included in each round.

### Stage 3: prioritisation of identified topics

The list of topics identified from the Delphi survey was presented to the TRiaDS Implementation Science Group (a multidisciplinary collaboration of international experts in implementation research) (Table S1) and the TRiaDS Professional Advisory Group [comprising the Assistant Director of Pharmacy (NES), an experienced community pharmacist, and a Policy and Development Pharmacist from Community Pharmacy Scotland] for discussion at the biannual TRiaDS meeting in December 2013. Topics were firstly mapped onto the Prescription for Excellence Strategy^21^ (for the purpose of assessment and reducing the number of topics); which were then further assessed based on the aim of the study (feasibility, acceptability, and potential impact for practice improvement). The topics selected at this meeting were then discussed with the Chief Pharmacist (Scotland) to seek final approval of the topic for further investigation within the time frame of TRiaDS‐P initiative.

### Ethical approval

The UK Governance Arrangements for Research Ethics Committees (GAfREC) made exemptions in 2011, and research involving NHS staff as participants by virtue of their professional role was excluded from the normal remit of NHS Research Ethics Committees.^19^ As such, ethical approval was not required for this study.

## Results

### Stage 1: key stakeholders: topic generation

Eight (a Director of Pharmacy, Lead Pharmacist of Primary & Community Care from a Health Board, Royal Pharmaceutical Society Practice and Policy Lead Scotland, a research lead for the pharmacy and prescribing support unit, a Policy & Development Pharmacist from Community Pharmacy Scotland, a Head of Pharmacy, a community pharmacist and a lay representative) of the 10 invitees attended the key stakeholder meeting. In total, 63 topics were generated which were then rationalised and combined, giving a final list of 20 topics.

### Stage 2: the Delphi survey

The Delphi survey was conducted over 6 weeks (October to December 2013). In total, 74 individuals (key stakeholders (*n* = 8/8, 100%); members of the wider stakeholder group (*n* = 10/31, 32%); and community pharmacists (*n* = 56/~2342, 0.2%) indicated interest in participating. Of these, 28 community pharmacists were respondents in Rounds 1 and 2 and 26 in Round 3 (Table 1), most of whom were women, aged 50–59 years and worked in large multiples as employee pharmacists (Table S2).

**Table 1:** Demographic characteristics of eDelphi respondents

Demographic data	Round 1	Round 2	Round 3
Number of respondents (*N*)	47	50	52
	**% (** ***n*** **)**	**% (** ***n*** **)**	**% (** ***n*** **)**
Gender			
Female	*63.8 (30)*	*58.0 (29)*	*57.7 (30)*
Male	27.7 (13)	28.0 (14)	26.9 (14)
Missing response	8.5 (4)	14.0 (7)	15.4 (8)
Age (years)			
18–29	10.6 (5)	8.0 (4)	7.7 (4)
30–39	6.4 (3)	10.0 (5)	7.7 (4)
40–49	36.2 (17)	28.0 (14)	26.9 (14)
50–59	*38.3 (18)*	*38.0 (19)*	*40.4 (21)*
>60	–	2.0 (1)	1.9 (1)
Missing response	8.5 (4)	14.0 (7)	15.4 (8)
Health board			
NHS Ayrshire and Arran	2.1 (1)	2.0 (1)	1.9 (1)
NHS Dumfries and Galloway	2.1 (1)	2.0 (1)	1.9 (1)
NHS Fife	4.3 (2)	2.0 (1)	1.9 (1)
NHS Forth Valley	8.5 (4)	10.0 (5)	7.7 (4)
NHS Grampian	17.0 (8)	14.0 (7)	13.5 (7)
NHS Greater Glasgow and Clyde	*25.5 (12)*	*24.0 (12)*	*26.9 (14)*
NHS Highland	2.1 (1)	6.0 (3)	5.8 (3)
NHS Lothian	15.0 (7)	8.0 (4)	7.7 (4)
NHS Shetland	2.1 (1)	2.0 (1)	1.9 (1)
NHS Tayside	10.6 (5)	12.0 (6)	9.6 (5)
NHS Borders	–	2.0 (1)	1.9 (1)
NHS Lanarkshire	–	2.0 (1)	1.9 (1)
NHS Western Isles	–	–	–
NHS Orkney	–	–	–
Missing response	10.6 (5)	14.0 (7)	17.3 (9)
Pharmacy sector			
Academic	8.5 (4)	8.0 (4)	5.8 (3)
Community	*59.6 (28)*	*56.0 (28)*	*50.0 (26)*
Primary care	19.1 (9)	18.0 (9)	1.9 (1)
Missing response	12.8 (6)	18.0 (9)	42.3 (22)
Type of community pharmacy			
Independent single outlet	14.9 (7)	12.0 (6)	13.5 (7)
Large multiple (>5 pharmacies)	*34.0 (16)*	*34.0 (17)*	*25.0 (13)*
Small multiple (2–5 pharmacies)	4.3 (2)	8.0 (4)	7.7 (4)
Other (self‐identified)	6.4 (3)	2.0 (1)	3.8 (2)
Employment status within community pharmacy			
Employee	*34.0 (16)*	*60.7 (17)*	*57.7 (15)*
Locum	6.4 (3)	14.3 (4)	11.5 (3)
Owner	15.0 (7)	21.4 (6)	23.1 (6)
Other	4.3 (2)	3.6 (1)	7.7 (2)

Maximum values are presented in italics.

#### Round 1

The 20 topics from Stage 1 were included in Round 1 (Table 2). In total, 47/74 (63.5%) individuals participated. Consensus was achieved with 17 topics, and the topics with the highest median score of 7.0 were as follows: *patient counselling for prescribed medication*;* promoting the appropriate sale and supply of over‐the‐counter (OTC) medicines*; and *promotion and delivery of the Minor Ailment Scheme (MAS)*.^1^ The 17 items were then added to Round 2 together with eight new topics identified by Round 1 participants (*pharmaceutical care of cancer; chronic obstructive pulmonary disease (COPD); asthma; dermatology; sexual health; improving medication adherence; pharmaceutical needs assessment; and pharmacovigilance*).

**Table 2:** Results of Round 1 eDelphi

Round 1 Topic heading	Mean (SD)	Interquartile range		
		25th	50th (median)	75th
1. Patients counselling for prescribed medication	6.44 (0.89)	6.00	*7.00*	7.00
2. Promoting the appropriate sale and supply of over‐the‐counter (OTC) medicines	6.24 (1.35)	6.00	*7.00*	7.00
3. Promotion and delivery of the Minor Ailment Scheme	5.98 (1.63)	5.00	*7.00*	7.00
4. Pharmaceutical care to promote medication adherence [e.g. monitored dosage system (MDS) assessment]	6.18 (0.93)	6.00	*6.00*	7.00
5. Effective use of community pharmacy workforce	6.09 (1.22)	6.00	*6.00*	7.00
6. Review of medication	5.91 (1.34)	5.00	*6.00*	7.00
7. Pharmaceutical care of diabetes	5.89 (1.09)	5.00	*6.00*	7.00
8. Community pharmacists' role in the reduction in medicines waste	5.80 (1.32)	5.00	*6.00*	7.00
9. Implementation of standard operating procedures (SOPs) (e.g. repeat dispensing, controlled drugs); national PGDs (e.g. repeat prescribing); and referral (e.g. direct referral out of hours) processes	5.78 (1.53)	5.00	*6.00*	7.00
10. Pharmaceutical care of cardiovascular disease	5.76 (1.20)	5.00	*6.00*	7.00
11. Pharmaceutical care of vulnerable patients (including high risk, sheltered housing residents, immigrants, homeless	5.73 (1.17)	5.00	*6.00*	7.00
12. Pharmaceutical care of chronic pain	5.71 (1.21)	5.00	*6.00*	7.00
13. Pharmaceutical care of drug misusers	5.69 (1.32)	5.00	*6.00*	7.00
14. Public Health Service: lifestyle behaviour services, e.g. weight management, alcohol screening, smoking cessation	5.69 (1.27)	5.00	*6.00*	7.00
15. Role of pharmacist prescribing	5.22 (1.59)	4.00	*6.00*	7.00
16. Public Health Service: screening services for risk or early disease detection, e.g. cancer, BP, and stroke	5.09 (1.44)	4.00	5.00	6.00
17. Pharmaceutical care of acute pain	5.04 (1.70)	4.50	5.00	6.00
18. Pharmaceutical care of acute dental problems	4.42 (1.53)	3.50	4.00	6.00
19. Antipsychotic medication use in dementia patients	4.22 (1.65)	3.00	4.00	5.00
20. Wound management	4.00 (1.31)	3.00	4.00	5.00

SD, Standard deviation.

Italics indicate topics included in Round 2.

#### Round 2

The response rate for Round 2 was 67.6% (50/74), with the majority (82.0%, *n* = 41) having taken part in Round 1. The topics (*n* = 23) included in this round and the results are presented in Table 3. Consensus was achieved with seven topics, three of which achieved a median score of 7.0: *promoting the appropriate sale and supply of OTC medicines; patient counselling for prescribed medication; and pharmaceutical care to promote medication adherence*. One of the seven prioritised topics was a duplicate, i.e. *pharmaceutical care to promote medication adherence, and improving medication adherence,* and was removed from the list leaving six topics for inclusion in Round 3.

**Table 3:** Results of Round 2 eDelphi

Round 2 Topic heading	Mean (SD)	Interquartile range		
		25th	50th (median)	75th
Promoting the appropriate sale and supply of over‐the‐counter (OTC) medicines [Guidance regarding effective strategies to promote the evidence‐based supply of OTC] [Round 1 median score = 7]	7.00 (0.00)	7.00	7.00	7.00
Patient counselling for prescribed medication [Guidance regarding strategies to be adopted by community pharmacists and/or the wider pharmacy team] [Round 1 median score = 7]	7.00 (0.00)	*7.00*	7.00	7.00
Pharmaceutical care of vulnerable patients (including high risk, sheltered housing residents, immigrants, homeless) [Guidance regarding the contribution which community pharmacists and/or the wider pharmacy team] [Round 1 median score = 6]	5.92 (1.19)	*6.00*	6.00	7.00
Pharmaceutical care to promote medication adherence [e.g. monitored dosage system (MDS) assessment] [Guidance regarding evidence‐based strategies to be adopted by community pharmacists and/or the wider pharmacy team to promote medication adherence] [Round 1 median score = 6]	7.00 (0.00)	*7.00*	7.00	7.00
Promotion and delivery of the Minor Ailment Scheme [Guidance regarding strategies for increasing the uptake and provision of the Minor Ailment] [Round 1 median score = 7]	6.60 (0.92)	*6.75*	7.00	7.00
Effective use of community pharmacy workforce [Guidance regarding making effective use of the pharmacy workforce to deliver safe, effective, and efficient community pharmacy services] [Round 1 median score = 6]	6.12 (0.87)	*6.00*	6.00	7.00
Improving medication adherence [Guidance regarding the contribution which community pharmacists and/or the wider pharmacy team can make to enhancing medication adherence] [Round 1 median score = 6]	6.32 (0.86)	*6.00*	7.00	7.00
Community pharmacist role in the reduction in medicines waste [Guidance regarding the contribution which community pharmacists and/or the wider pharmacy team] [Round 1 median score = 6]	5.98 (0.82)	5.00	6.00	7.00
Implementation of standard operating procedures (SOPs) (e.g. repeat dispensing, controlled drugs); national patient group directions (PGDs) (e.g. repeat prescribing), and referral (e.g. direct referral out of hours) [Round 1 median score = 6]	5.96 (1.06)	5.00	6.00	7.00
Review of medication [Guidance regarding safe and effective individual reviews of patient medication] [Round 1 median score = 6]	5.98 (1.18)	5.00	6.00	7.00
Pharmaceutical care of cardiovascular disease [Guidance regarding the contribution which community pharmacists and/or the wider pharmacy team] [Round 1 median score = 6]	5.74 (0.82)	5.00	6.00	6.00
Pharmaceutical care of chronic pain [Guidance regarding the contribution which community pharmacists and/or the wider pharmacy team] [Round 1 median score = 6]	5.62 (1.24)	5.00	6.00	6.00
Pharmaceutical care of diabetes [Guidance regarding the contribution which community pharmacists and/or the wider pharmacy team] [Round 1 median score = 6]	5.76 (0.82)	5.00	6.00	6.00
Pharmaceutical care of drug misusers [Guidance regarding the contribution which community pharmacists and/or the wider pharmacy team can make to the management and support of patients who are drug misusers] [Round 1 median score = 6]	5.76 (1.00)	5.00	6.00	6.00
Public Health Service: lifestyle behaviour services, e.g. weight management, alcohol screening, smoking cessation [Guidance regarding the contribution which community pharmacists and/or the wider pharmacy team] [Round 1 median score = 5]	5.72 (1.29)	5.00	6.00	7.00
Role of pharmacist prescribing [Guidance regarding the effective and efficient use of community pharmacist prescribers] [Round 1 median score = 6]	5.60 (1.34)	5.00	6.00	7.00
Pharmaceutical care of cancer [Guidance regarding the contribution which community pharmacists and/or the wider pharmacy team can make to the care of patients with a diagnosis of cancer]	5.64 (0.85)	5.00	6.00	6.00
Pharmaceutical care of chronic obstructive pulmonary disease (COPD) [Guidance regarding the contribution which community pharmacists and/or the wider pharmacy team can make to the care of patients with a diagnosis of COPD]	5.78 (0.70)	5.00	6.00	6.00
Pharmaceutical care of asthma [Guidance regarding the contribution which community pharmacists and/or the wider pharmacy team can make to the care of patients with a diagnosis of asthma]	5.92 (0.75)	5.00	6.00	6.00
Pharmaceutical care of dermatology [Guidance regarding the contribution which community pharmacists and/or the wider pharmacy team can make to the care of patients with dermatological conditions]	5.70 (0.93)	5.00	6.00	6.00
Pharmaceutical care of sexual health [Guidance regarding the contribution which community pharmacists and/or the wider pharmacy team can make to the care of patients and/or pharmacy users in terms of promoting and maintaining good sexual health]	5.54 (0.97)	5.00	6.00	6.00
Pharmaceutical care needs assessment [Guidance regarding the contribution which community pharmacists and/or the wider pharmacy team can make to planning, conducting, or supporting pharmaceutical care needs assessment]	5.90 (1.14)	5.00	6.00	7.00
Pharmacovigilance [Guidance regarding the contribution which community pharmacists and/or the wider pharmacy team can make to monitoring and managing the safety and risks of medicines]	5.56 (1.54)	5.00	6.00	7.00

SD, Standard deviation.

Italics indicate topics included in Round 3.

#### Round 3

The Round 3 response rate was 70.3% (52/74); 90.4% (*n* = 47) of these participants participated in Rounds 1 and 2. All six topics achieved highest score of median (7.0) and interquartile range (IQR = 7) (Table 4) and indicated the equal importance of these topics. Of these topics, four (*promoting the appropriate sale and supply of OTC medicines; patient counselling for prescribed medication; pharmaceutical care to promote medication adherence; and promotion and delivery of the Minor Ailment Scheme*) had a median score of 7.0 and two (*pharmaceutical care of vulnerable patients; effective use of community pharmacy workforce*) had a median score of 6.0 in Round 2.

**Table 4:** Results of Round 3 eDelphi

Round 3 Topic heading	Mean (SD)	Interquartile range		
		25th	50th (median)	75th
Promoting the appropriate sale and supply of OTC medicines [Round 1 median score = 7 and Round 2 median score = 7]	7.00 (0.00)	7.00	7.00	7.00
Patient counselling for prescribed medication [Round 1 median score = 7 and Round 2 median score = 7]	7.00 (0.00)	7.00	7.00	7.00
Promotion and delivery of the Minor Ailment Scheme [Round 1 median score = 7 and Round 2 median score = 7]	7.00 (0.00)	7.00	7.00	7.00
Pharmaceutical care to promote medication adherence [e.g. monitored dosage system (MDS) assessment] [Round 1 median score = 6 and Round 2 median score = 7]	7.00 (0.00)	7.00	7.00	7.00
Pharmaceutical care of vulnerable patients (including high risk, sheltered housing residents, immigrants, homeless) [Round 1 median score = 6 and Round 2 median score = 6]	7.00 (0.00)	7.00	7.00	7.00
Effective use of community pharmacy workforce [Round 1 median score = 6 and Round 2 median score = 6]	7.00 (0.00)	7.00	7.00	7.00

### Stage 3: prioritisation of identified topics

All six topics were assessed at the TRiaDS meeting. The topic *effective use of community workforce* cuts across all priority topics and therefore, it was proposed to be incorporated into each of the priority topics selected to undergo the TRiaDS process. The topics *pharmaceutical care for medication adherence* and *patient counselling for prescribed medication* were combined, because it was believed that any interventions developed to influence counselling would ultimately seek to achieve enhanced medication adherence. Finally, the four topics (*promoting the appropriate sale and supply of OTC medicines, promotion and delivery of the MAS, pharmaceutical care to promote medication adherence via patient counselling,* and *pharmaceutical care of vulnerable patients*) were prioritised based on the Prescription for Excellence Strategy and were further assessed based on the aim of this study. The topic selected for the next stage of study, following the negotiation stage with the chief pharmacist, was *promoting the appropriate sale and supply of OTC medicines*.

## Discussion

A wide range of areas for improvement was generated. The eDelphi demonstrated consistency of views with all six priority topics from Round 3 achieving high agreement. The priority topic selected for the next part of TRiaDS‐P, promoting the appropriate sale and supply of OTC medicine, met all criteria in terms of priorities and therefore, it was found to be the most important topic at the time based on the WHICH^20^ and Prescription for Excellence guidance.^21^

Engagement with stakeholders, as well as the Professional Advisory Group and experts in implementation research, ensured that both views of the pharmacy profession in Scotland and multidisciplinary scientific expertise were taken into account in the process. Identifying service‐driven priorities for practice improvement is an essential starting point for implementation research. To our knowledge, this is the first prioritisation study to identify topics for community pharmacy practice improvement and guideline development. Comprehensive engagement with stakeholders identified service‐driven priorities. For example, the eDelphi component was open to any community pharmacist within the NES database who wished to participate. All but two of the 14 Health Board areas in Scotland were represented in each round (Table 1). However, no responses were received from individuals in the most remote Health Boards (NHS Orkney and NHS Western Isles) which had seven community pharmacies (representing 0.6% of the total number of pharmacies in Scotland) at the time of the survey.

Not all the potential participants who initially agreed to participate in the eDelphi did so. Whilst the overall participation by community pharmacists was low, the study was successful in obtaining high response rates across the three rounds of Delphi survey, with the response rate increasing with each eDelphi round. Whilst the demographic profile of pharmacist respondents in our study is similar to a survey of registered pharmacy professionals conducted in 2013 by the General Pharmaceutical Council (GPhC) in terms of gender, employment status, and type of pharmacy, i.e. large multiple,^22^ older pharmacists were over‐represented in our study and as such, may have influenced the results derived.^23^ The highest numbers of community pharmacy respondents were located in NHS Health Boards Greater Glasgow and Clyde, Lothian, and Grampian, which also have the highest numbers of community pharmacies. However, the percentage of pharmacists who responded to eDelphi was comparable with the percentage of community pharmacists working in different health boards of Scotland (Table S3).^24^

The maximum number of participants in any round was 52 despite 74 individuals initially indicating their willingness to participate. The reason for the subsequent non‐response of individuals was not explored. We can only speculate about the causes of non‐response. For example, they might have misinterpreted the purpose of this study or perhaps considered the survey too long or time‐consuming to complete. Only one lay representative attended Stage 1. The purpose of this study was to identify service improvement priorities from the pharmacy profession's perspective and as such, no further lay representatives were involved. Involvement of lay representatives in other stages of this study could have changed the outcome of the prioritisation exercise.

The current strategy for Pharmaceutical Care in Scotland, *Prescription for Excellence,* (http://www.gov.scot/resource/0043/00434053.pdf) was published in September 2013, i.e. after the key stakeholder meeting but prior to the eDelphi process. Its publication is likely to have influenced the content and outcome of this process. It is likely that priorities identified now may differ from those identified by this exercise. However, most were included in this strategy document which continues to inform pharmacy practice in Scotland and as such, they remain relevant.

### Promoting the appropriate sale and supply of over‐the‐counter medicines

It is likely that one of the main drivers for the identification of ‘promoting the appropriate sale and supply of OTC medicines’ as a priority topic was the publication of a *Which?* report earlier in 2013, titled ‘Are some pharmacies failing?’.^20^ The report was published several months prior to the prioritisation exercise. As with earlier *Which?* reports, sub‐optimal practice was identified across pharmacies in relation to the sale and/or supply of different OTC requests. This demonstrates the sensitivity of this type of prioritisation exercise to high‐profile topics within current public and/or professional awareness. However, sub‐optimal practice with this service has been demonstrated previously and consistently with ‘academic’ studies,^25^, ^26^, ^27^, ^28^, ^29^ confirming that this service warrants further attention in terms of quality improvement. Furthermore, there is substantial evidence to suggest that the extent of information exchange (communication) during consultations for OTC medicines is a major factor affecting the appropriateness of these consultations.^30^, ^31^, ^32^, ^33^, ^34^, ^35^ To date, however, there has been no in‐depth theory‐driven exploration of the key determinants of pharmacist and pharmacy staff behaviour in terms of communication performance in general and with information elicitation in particular, during the management of these OTC consultations. As such, the next stage of the *TRiaDS‐P* programme will be the exploration of the barriers and facilitators associated with this behaviour using semi‐structured interviews with pharmacists and MCAs. The interviews will be underpinned by the Theoretical Domains Framework (TDF)^36^ and the capability, opportunity, and motivation‐behaviour (COM‐B) system,^37^ the results of which will be mapped using the Behaviour Change Wheel^38^ to identify potential interventions for change.

### Other priorities

The five remaining priority topics that were identified will also require further exploration and development. The ‘pharmaceutical care of vulnerable patients’ and the ‘effective use of [the] community pharmacy workforce’ were both identified in Prescription for Excellence and as such, are less likely to be included in the *TRiaDS‐P* programme because they will receive national attention and development.

Patient counselling for prescribed medication is not entirely dissimilar to the overall priority topic. Effective consultation management, whether for OTC or prescribed medicines, relies upon effective communication behaviour and consultation skills.^39^ In 2011, the New Medicines Service was introduced in England to improve medication adherence of patients with long‐term conditions.^40^ No similar service exists in Scotland (or to our knowledge in any other country).

The Promotion and Delivery of the Minor Ailment Scheme (eMAS) was introduced in Scotland in 2006, with the purpose of providing equity of access to treatment and advice for common conditions and as a strategy for reducing demand on higher cost service providers, e.g. general practitioners (GPs).^41^ However, there is evidence to suggest that the uptake of eMAS in Scotland has been lower than anticipated with figures suggesting that not all eligible individuals have registered.^42^ However, this topic was not selected for reasons of political sensitivity and an ongoing review of the service.

## Conclusions

This prioritisation study was the first step in the *TRiaDS‐P* programme and adopted a systematic, inclusive, and rapid (<5 months) approach to identify priorities for community pharmacy practice improvement in Scotland. By taking a systematic, stepped approach underpinned by theory to the identification, development, and evaluation of potential educational, service and policy interventions for *promoting the appropriate sale and supply of OTC medicines*,* TRiaDS‐P* increases the likelihood of the efficient and effective translation of knowledge into community pharmacy practice for improved patient care. This methodology could be used by other disciplines and in other countries to prioritise and address their quality improvement agenda.

## Declarations

### Conflict of interest

The Author(s) declare(s) that they have no conflicts of interest to disclose.

### Funding

This work was supported by the NHS Education for Scotland.

### Acknowledgements

We thank all the participants in stages 1 and 2, members of the TRiaDS Implementation Science Group, and Professional Advisory Group members for their guidance and contribution to the study. We also thank Ms. Trish Graham for providing invaluable administrative support for this study.

### Authors' contribution

MW contributed to the conceptual and theoretical development of the study and is the Research Lead for the TRiaDS‐Pharmacy programme. RN and MW drafted the manuscript. All authors critically reviewed and contributed to draft revisions, and read and approved the final version of the manuscript.

## Supporting information


**Table S1.** List of TRiaDS Implementation Science Group members.
**Table S2.** Demographic characteristics of community pharmacist respondents.
**Table S3.** Percentage of community pharmacists working in different health boards of Scotland and percentage who responded to the eDelphi survey.

## Note

1 Pharmacy‐based minor ailment schemes (MAS) provide public access to NHS treatment and/or advice (e.g. acne, diarrhoea) via a pharmacist or pharmacy personnel, or, where appropriate, to onward referral to other health professionals.^43^
