# Supplementary material for: Quality improvement of community pharmacy services: a prioritisation exercise
Source: Int J Pharm Pract. 2017 Mar 27;26(1):39–48. doi: 10.1111/ijpp.12354 (PMC5811898; doi:10.1111/ijpp.12354)
Supplement: Supplementary file 1 — Table S1. List of TRiaDS Implementation Science Group members. Table S2. Demographic characteristics of community pharmacist respondents. Table S3. Percentage of community pharmacists working in different health boards of Scotland and percentage who responded to the eDelphi survey. [file IJPP-26-39-s001.docx]

Table S1. List of TRiaDS Implementation Science Group members

| TRiaDS Implementation Science Group members |
| --- |
| Professor Sandra Eldridge, Professor of Biostatistics/Director Pragmatic Clinical Trials Unit, Centre for Primary Care and Public Health, Blizard Institute, Barts and the London Medical School of Medicine and Dentistry, England. |
| Professor Robbie Foy, Professor of Primary Care, Leeds Institute of Health Sciences,  University of Leeds, England. |
| Professor Jeremy Grimshaw, Professor Dept of Medicine, Centre for Practice-Changing Research, Ottawa Hospital Research Institute, Canada. |
| Professor Lorna McKee, Professor of Management and Programme Director of the Delivery of Care Programme, Health Services Research Unit, University of Aberdeen, Scotland. |
| Professor Carole Torgerson, Professor, School of Education, Durham University, England. |
| Professor Shaun Treweek, Professor of Health Services Research, Health Services Research Unit, University of Aberdeen, Scotland. |
| Professor Michel Wensing, Professor of Implementation Science, Faculty of Medical Sciences  Radboud University Medical Centre, the Netherlands. |
| Dr Debbie Bonetti, Senior Research Fellow, Dental Health Services Research Unit, University of Dundee, Scotland. |

Table S2. Demographic characteristics of community pharmacist respondents

| **Demographic data** | **Round 1** | **Round 2** | **Round 3** |
| --- | --- | --- | --- |
| ***Number* of *respondents (N)*** | 28 | 28 | 26 |
|  | **% (n)** | **% (n)** | **% (n)** |
| ***Gender***  Female | **67.9 (19)** | **60.7 (17)** | **61.5 (16)** |
| ***Age (years)***  18-29  30-39  40-49  50-59  >60 | 14.3 (4)  3.6 (1)  32.1 (9)  **50.0 (14)**  - | 14.3 (4)  10.7 (3)  25.0 (7)  **50.0 (14)**  - | 11.5 (3)  7.7 (2)  26.9 (7)  **53.9 (14)**  - |
| ***Health Board***  NHS Ayrshire and Arran  NHS Dumfries and Galloway  NHS Fife  NHS Forth Valley  NHS Grampian  NHS Greater Glasgow and Clyde  NHS Highland  NHS Lothian  NHS Shetland  NHS Tayside  NHS Borders  NHS Lanarkshire  NHS Western Isles  NHS Orkney | 3.6 (1)  3.6 (1)  7.1 (2)  10.7 (3)  14.3 (4)  **25.0 (7)**  3.6 (1)  17.9 (5)  3.6 (1)  10.7 (3)  -  -  -  - | 3.6 (1)  3.6 (1)  3.6 (1)  14.3 (4)  10.7 (3)  **25.0 (7)**  10.7 (3)  10.7 (3)  3.6 (1)  14.3 (4)  -  -  -  - | 3.8 (1)  3.8 (1)  3.8 (1)  15.4 (4)  15.4 (4)  **23.1 (6)**  11.5 (3)  7.7 (2)  3.8 (1)  11.5 (3)  -  -  -  - |
| ***Type of community pharmacy***  Independent Single Outlet  Large multiple (>5 pharmacies)  Small multiple (2–5 pharmacies)  Other (self-identified) | 25.0 (7)  **57.1 (16)**  7.1 (2)  10.7 (3) | 21.4 (6)  **60.7 (17)**  14.3 (4)  3.6 (1) | 26.9 (7)  **50.0 (13)**  15.4 (4)  7.7 (2) |
| ***Employment Status*** Employee  Locum  Owner  Employee  Other | 10.7 (3)  25.0 (7)  **57.1 (16)**  7.1 (2) | 14.3 (4)  21.4 (6)  **60.7 (17)**  3.6 (1) | 11.5 (3)  23.1 (6)  **57.7 (15)**  7.7 (2) |

Table S3. Percentage of community pharmacists working in different health boards of Scotland and percentage who responded to the eDelphi survey

| ***Health Board*** | Round 1 | Round 2 | Round 3 | % Community pharmacists in different health boards |
| --- | --- | --- | --- | --- |
| NHS Ayrshire and Arran  NHS Dumfries and Galloway  NHS Fife  NHS Forth Valley  NHS Grampian  NHS Greater Glasgow and Clyde  NHS Highland  NHS Lothian  NHS Shetland  NHS Tayside  NHS Borders  NHS Lanarkshire  NHS Western Isles  NHS Orkney | 3.6  3.6  7.1  10.7  14.3  25.0  3.6  17.9  3.6  10.7  -  -  -  - | 3.6  3.6  3.6  14.3  10.7  25.0  10.7  10.7  3.6  14.3  -  -  -  - | 3.8  3.8  3.8  15.4  15.4  23.1  11.5  7.7  3.8  11.5  -  -  -  - | 7.8  2.7  6.7  6  10.4  23.2  6.4  14.5  0.4  7.3  2.3  11.4  0.2  0.3 |
